# Anti-IL5 Drugs in COVID-19 Patients: Role of Eosinophils in SARS-CoV-2-Induced Immunopathology

**DOI:** 10.3389/fphar.2021.622554

**Published:** 2021-03-09

**Authors:** Daniele Pala, Marco Pistis

**Affiliations:** ^1^ Unit of Clinical Pharmacology, University Hospital Agency of Cagliari, Cagliari, Italy; ^2^ Department of Biomedical Sciences, Division of Neuroscience and Clinical Pharmacology, University of Cagliari, Cagliari, Italy; ^3^ Neuroscience Institute, National Research Council of Italy (CNR), Section of Cagliari, Cagliari, Italy

**Keywords:** COVID-19, asthma, type 2 response, eosinophils, interleukin-5, anti-IL5 drugs

## Abstract

SARS-CoV-2 infection stimulates a complex activation of the immune system. Eosinophils belong to the host’s defense equipment against respiratory viruses. In the first phase of the infection, eosinophils contribution is probably appropriate and beneficial, as they facilitate the suppression of the viral replication. However, in severe COVID-19 patients, during the second and third phases of the disease, eosinophils may participate in a maladaptive immune response and directly contribute to immunopathology. In fact, in severe patients, the immune response is prevalently T helper 1 type, but T helper 2 is also present. Eosinophils’ expansion and activation are stimulated by Type 2 cytokines, especially IL-5. Moreover, bronchial asthma, in which eosinophils play a central role, seems not to be a major risk factor for severe COVID-19. Among possible explanations, asthmatic patients are often treated with corticosteroids, which have been demonstrated to reduce the progression to critical COVID-19 in hospitalized patients. In addition to steroids, severe asthmatic patients are currently treated with biological drugs that target Type 2 immune response. Because IL-5 is necessary for the growth, survival, and activation of eosinophils, IL-5 inhibitors, such as mepolizumab, decrease the peripheral blood count of eosinophils, but do not influence eosinophils activation in the airway. In severe COVID-19 patients, the blockade of eosinophils’ activation might contrast harmful immunity.

## Introduction

COVID-19 pandemic is a grave challenge for every health care professional worldwide. A better understanding of disease pathogenesis might boost a more effective and targeted therapy, with the hope of saving as many lives as possible. Here, a rationale for anti-IL5 drug use in severe COVID-19 patients is discussed. Since the beginning of the Health Emergency, several drugs, mostly antiviral and anti-inflammatory, have been repurposed and a vast number of clinical trials started worldwide to assess their efficacy and safety with an unprecedented speed. However, to date, only systemic corticosteroids have been demonstrated to prevent deaths in COVID-19 most severe patients and received formal approval from Regulatory Agencies (Sterne et al., 2020).

One of the most relevant findings regarding COVID-19’s natural history highlights three different clinical stages, each requiring different types of therapies. In the first stage, the principal feature is viral replication, which can be contrasted by antiviral drugs, such as remdesivir. In the second stage, the pulmonary one, clinical symptoms become more prominent because of the beginning of the host’s immune response. The third stage is characterized by an immunopathologic response, which can result in a cytokine storm in the most severe cases (Siddiqi and Mehra, 2020). In the second and third stages, immunotherapies can be indicated (Sandkovsky et al., 2020).

Mounting evidence shows that COVID-19’s immunopathology has peculiar features. It has been highlighted that the classical T helper 1 (Th1) response is defective during acute infection, but it is prevalent among memory T cells in convalescent individuals (Sekine et al., 2020). Patients with moderate COVID-19 experience lower signs of inflammatory activation in comparison with severe COVID-19 patients. The inflammation follows an initial increment of cytokines, and subsequently a decrease of type 1 and type 3 responses (Lucas et al., 2020). The peripheral blood count of CD4^+^ and CD8^+^ T cells in terms of absolute number and frequency display a significant reduction in patients with either moderate or severe infection (Sekine et al., 2020). Nevertheless, Th1 CD4^+^, Th1 CD8^+^, and Natural Killer T cells are activated to promote antiviral activity and drive the disease recovery in moderate infection (Zhang J. Y. et al., 2020). Finally, in convalescent individuals, other authors analyzed SARS-CoV-2-specific memory T cells and found that CD4^+^ T cells mainly produced Interferon-γ (IFN- γ), Interleukin-2 (IL-2), and Tumor Necrosis Factor- α (TNF-α), whereas CD8^+^ T cells mainly produced IFN-γ (Sekine et al., 2020).

## Th2 Immune Response in Severe Patients

The first reports showed a significant difference in T helper 2 (Th2) cytokines in severe COVID-19 patients hospitalized into Intensive Care Units, especially IL-4 and IL-10, providing initial evidence in favor of Th2 activation, but significant differences in IL-5 levels were not found (Huang C. et al., 2020).

In severe patients, the immune system activation is characterized by a distorted interferon production and a disordered T cellular response that lead to profound immune exhaustion and broad T cell expansion (Zhang J. Y. et al., 2020). Patients with severe COVID-19 produce elevated levels of cytokines during the clinical course of the disease. These patients showed a pattern of Th1 activation, but also showed a Type 2 immune response, characterized by an increase of IL-5, IL-13, eotaxin-2, immunoglobulin E (IgE), and eosinophils. Type 2 biomarkers remain elevated in patients with severe COVID-19 and correlate with the worst course of the disease. Levels of eosinophils were significantly higher in patients with severe COVID-19 than those with a moderate disease or healthy controls (Lucas et al., 2020).

Similarly, levels of IL-5 were higher in severe patients than in those with a moderate disease or healthy control. IL-13 differs in severe COVID-19 compared to controls. In patients with severe disease, IgE immunoglobulins slightly increased during the disease course (Lucas et al., 2020).

Conversely, IL-4 did not diverge between the two groups (Lucas et al., 2020). The last result is confirmed by another observational study (Mann et al., 2020). Nevertheless, IL-4, IL-5, and IL-13 displayed a trend toward an increase in the clinical scenario of severe COVID-19, and a reason for the interest is the importance of IL-5 to predict mortality, with a predictive value of around 0.73 (Lucas et al., 2020).

Elsewhere, eotaxins, a group of chemokines involved in the chemotaxis of eosinophils, revealed conflicting results: in the previous study, eotaxin-1 and eotaxin-3 were reduced in COVID-19 (Lucas et al., 2020) similarly to another immunophenotyping study in which eotaxin-1 was reduced (Mathew et al., 2020). Differently, eotaxin-2 was increased as compared to controls (Lucas et al., 2020). The eosinophil count was similar and within the normal values range in the two studies: a mean count of 100 cells/μL with a maximum of 250 cells/μL in the first (Lucas et al., 2020) and count below 100 cells/μL with a maximum at 400 cells/μL, in the second (Mathew et al., 2020). However, the two studies agree with the increase of fundamentals type 2 cytokines, like IL-5 and IL-13 in severe COVID-19 patients, at least in a subset of them (Lucas et al., 2020; Mathew et al., 2020). Another immunophenotyping study shows decreasing participation of eosinophils from mild to severe groups (Mann et al., 2020).

The specific activation of the type 2 immunity has been confirmed by different groups. Roncati et al. (2020) show that in all the 15 peripheral blood samples from intensive care COVID-19 patients, cytological signals of Th2 immune response were found, namely eosinophilia, basophilia, degranulated eosinophils, and plasma cells (Roncati et al., 2020). In general, stimulation of SARS-CoV-2-specific T cells from peripheral blood of severe COVID-19 patients drives a prevalent production of Th1 cytokines (IFN-γ, TNF-α, IL-2), but also Th2 (IL-5, IL-13, IL-9, IL-10) and Th17 (IL-17A, IL-17F, and IL-22) cytokines were detected (Weiskopf et al., 2020). The specific T-cell response against SARS-CoV-2 was assessed by stimulating peripheral blood cells with the Spike protein and other viral peptides. Mononuclear blood cells from COVID-19 vs. non-COVID-19 cells produced a significant higher number of cytokines such as IL-2 (50.08 vs. 0), IFN-γ (90.16 vs. 0), IL-4 (0.52 vs. 0), IL-13 (0.84 vs. 0) and MCP-1 (4,602 vs 359.2), among which IL-4 and IL-13 are key Th2 mediators (Petrone et al., 2020). The involvement of IL-4 and IL-13 was confirmed by others, who found a relative gene expression upregulation in CD4^+^ T-cells from COVID-19 patients by using a single cell transcriptomics approach (Kalfaoglu et al., 2020).

In a single-cell analysis, six subtypes of CD4^+^ T cell clusters have been characterized. In particular, 2 T CD4^+^ effector subtypes, CD4+-GZMK (granzyme) and CD4+-GNLY (granulysin) have been found. CD4+-GNLY cells displayed a high production of TBX21; consequently, they were Th1-like cells. Conversely, CD4+-GZMK and CD4+memory cells revealed Th2-like features with high production of GATA3 (Zhang J. Y. et al., 2020).

To evaluate the differences between peripheral blood and bronchoalveolar lavage fluid (BALF) in COVID-19 patients, mononuclear cells from both compartments were compared. A clonal increase of Th1, Th2, and Th17 cells was found in severe cases. A significant difference has been observed between matched BALF and plasma samples for IL-5, IL-8, IL-17, and INF-α, with higher levels of these cytokines in the BALF (Xu et al., 2020).

The timing analysis is a critical point to consider when observing the relative increase or decrease of a single cytokine or a cell subset. In a longitudinal analysis of a fatal case, IL-5 was found increased between 1- and 2-times on the 14th day since the infection, but showed a decrease between day 16 and day 22 and a further increase on day 24 (Bouadma et al., 2020). This pattern is in line with observations from a large-scale study that show an increase of IL-5 levels within days 6–11 from symptom onset, to which a subsequent increase of eosinophils follows on days 11–15, and a simultaneous slowdown of IL-5 rise on days 11–15. The last phase is characterised by a further increase of IL-5 on days 16–20 and a relative slowdown of blood eosinophil count on days 16–20 (Lucas et al., 2020). Finally, deceased patients had higher levels of IL-5 than patients with moderate or severe disease (Liu et al., 2020).

Probably, there are many explanations for these observations. In the first and early second phases of SARS-CoV-2 infection, eosinophils can contribute to the elimination of the virus, thanks to the antiviral activity of their enzymes. Later, during the advanced second phase of COVID-19, when the immune system starts slowing down viral replication, their antiviral properties are not requested, so IL-5 production is moderately reduced (Bouadma et al., 2020; Lucas et al., 2020). Nevertheless, eosinophils enrollment during the second phase may contribute to harm target tissues and progress the pathology. In the last phase of a severe course, the immune system undertakes a pathologic pathway characterized by a broad and uncontrolled cytokines storm, with a new pathological increase of IL-5 (Lucas et al., 2020; Bouadma et al., 2020; Liu et al., 2020).

## Role of Eosinophils in Pathology

Eosinophils play an important role in protecting the host against viral infections. They recognize viruses through Toll-like receptors (Flores-Torres et al., 2019). Eosinophils participate in the antiviral immune response because of their preformed granules, which contain cytotoxic proteins, such as eosinophil peroxidase, major basic protein, and 2 RNases (eosinophil neurotoxin and eosinophil cationic protein) (Ramirez et al., 2018; Flores-Torres et al., 2019). They produce reactive nitrogen species with antiviral activity (Flores-Torres et al., 2019).

Eosinophils protect the host from respiratory viruses, such as respiratory syncytial virus, rhinovirus, parainfluenza, and influenza virus (Ramirez et al., 2018). Eosinophils can rapidly internalize and inactivate respiratory syncytial virus and influenza virus, an ability that is compromised in asthma, with a close correlation with asthma exacerbation (Sabogal Piñeros et al., 2019a). Influenza A virus stimulates pulmonary lymphoid cells to generate large amounts of IL-5, which attracts eosinophils in the respiratory tissues (Gorski et al., 2019). In response to the influenza A virus, eosinophils activate, undergo degranulation, and act as antigen-presenting cells, then induce CD8^+^ T cell effector functions (Samarasinghe et al., 2017). A comprehensive review of eosinophils and viral infections was made by Flores-Torres et al. (2019).

Toxic proteins and mediators released from activated eosinophils participate in the pathogenesis of asthma and other allergic and immune-mediated diseases (Ramirez et al., 2018). Asthma exacerbations can usually be triggered by viral infections (Flores-Torres et al., 2019). During asthma exacerbations, eosinophils are activated to release free eosinophil granules and undergo lysis (Muniz-Junqueira et al., 2013). In theory, two principal ways of dying exist for eosinophils: primary lysis and apoptosis. Surprisingly, some signals that induce eosinophils apoptosis, lead to cell lysis (Persson and Uller, 2013). Activated eosinophils from asthma and allergic diseases express on surface sialic acid-binding immunoglobulin-like lectin (Siglec)-8. Siglec-8 normally causes cell death, but in presence of IL-5, it induces ROS-dependent cell death, characterized by necrotic features and granules release (Kano et al., 2013). The characteristic of undergoing primary lysis clarify because apoptotic eosinophils have not been found yet in affected tissues from different eosinophilic diseases (Persson and Uller, 2013; Persson and Uller, 2014). Corticosteroids reduce eosinophilic granules in the sputum of asthma exacerbation and probably do anti-IL-5 drugs (Persson and Uller, 2014).

Primary lysis of eosinophils is characterised by cell membrane rupture, a subsequent release of free eosinophilic granules content, and damage-associated molecular patterns (DAMPs) from cytoplasm and nucleus (Persson and Uller, 2014). DAMPs, like ATP, High mobility group box 1 protein (HMGB1), RNAs, DNAs, and IL-1β stimulate a potent activation of inflammation. Through DAMPs signaling, a dying cell recruits phagocytes, like macrophages, dendritic cells, and epithelial cells. In turn, phagocytes, which have pattern recognition receptors (PRRs), start eating irreversibly damaged cells through a process called efferocytosis. Activated phagocytes by a necrotic dying cell produce pro-inflammatory cytokines such as IL-1, IL-6, and IL-12 (Kolb et al., 2017).

Interestingly, regarding COVID-19 pathology, phagocytes and related cytokines (especially IL-1 and IL-6) have been recognised to play a central role (Bonaventura et al., 2020). A similarity between COVID-19 and secondary Hemophagocytic lymphohistiocytosis (HLH) syndrome has been proposed (Mehta et al., 2020). HLH is characterized by ungoverned growth and activation of phagocytes. HLH is often triggered by viral infections, like herpes viruses (Epstein-Barr virus and cytomegalovirus mainly), H1N1 influenza virus, parvovirus B19, HIV, or other viruses. From a haematological point of view, HLH is characterized by leukopenia (Ramos-Casals et al., 2014).

A suggestive hypothesis is that, in COVID-19, eosinophils stimulated by SARS-CoV-2 infection would migrate to the lungs and undergo primary lysis, which in turn recruit phagocytes. In a severe patient, uncontrolled phagocyte activation causes hyper inflammation and a cytokine storm. The interplay between lung eosinophils and SARS-CoV-2 needs more in-depth analysis, considering potential therapeutic implications.

## Eosinophils Involvement in COVID-19

Since the first laboratory reports of COVID-19 severe patients, the peripheral blood count of circulating eosinophils is mostly found below the normal value range (Zhang Z. L. et al., 2020), and there is a significant difference between moderate vs. critical disease (Liao et al., 2020). These observations have been confirmed by many authors and by different meta-analyses (Danwang et al., 2020; Ghahramani et al., 2020).

Since the first phases of the infection, patients may undergo an active migration of circulating eosinophils from the peripheral blood to target tissues, because of their antiviral functions (Flores-Torres et al., 2019). In the subsequent phases, peripheral eosinophils start declining. An explanation of the observed eosinopenia in the last phases of COVID-19 disease would consider the nearly concomitant increase of eosinophils-stimulating cytokines, such as IL-5 and GM-CSF, at least in a subset of patients (Lucas et al., 2020; Mathew et al., 2020), and the complex interactions with other actors of the immune system. A possible explanation is the induction of eosinophils apoptosis caused by endogenous or therapeutic glucocorticoids (Ilmarinen et al., 2014). Also, cytokines such as IFN-α and IFN-γ (type 1 IFNs) can induce eosinophils apoptosis (Morita et al., 1996). Nevertheless, in COVID-19 type 1 IFNs production is limited (Acharya et al., 2020). Another explanation contemplates the induction of cell primary lysis (Persson and Uller, 2013). The last hypothesis has much more therapeutic implications because of the stimulation of efferocytosis and inflammation from primary lytic eosinophils (Persson and Uller, 2014).

During hospitalization, eosinophils’, lymphocytes’, and platelets’ count showed a different pattern in the survivors’ peripheral blood compared to the non-survivors: in survivors, the cell count increased progressively, whereas, in the non-survivor, it maintained low levels and finally declined. The laboratory eosinophils count is a negative prognostic factor for non-survivors, specifically eosinophils on hospital admission less than 0.03 × 10^9^/L (HR, 2.12; 95% CI, 0.91–4.98), whereas eosinophils (x10^9^/L) > 0.05 vs ≤ 0.05 was a protective factor for fatal outcome (HR, 0.38; 95% CI, 0.17–0.83). In Kaplan-Meier analysis, the survival was significantly higher in patients with eosinophils >0.05 (x10^9^/L) compared to those with eosinophils ≤0.05 (x10^9^/L) (Chen et al., 2020). Blood eosinophils showed a positive correlation with lymphocytes in severe and non-severe patients after admission (Zhang J. J. et al., 2020).

The laboratory monitoring of peripheral eosinophil count has been proposed as a precision tool to monitor the clinical course of the disease and predict the admission to Intensive Care Unit (ICU) (Huang J. et al., 2020). The eosinophils count = 0 (×10^9^ per L) predicts the admission to ICU with a mean sensitivity of 48.15 (95% CI: 28.7–68.1) and a mean specificity of 98.88 (95% CI: 93.9–100.0), a positive predictive value of 92.9 (95% CI: 66.1–99.8) and a negative predictive value of 86.3 (95% CI: 78.0–92.3). In the ROC analysis, the AUC of eosinophils is 0.763 (95%CI: 0.641–0.886) (Sun et al., 2020). The very high value of specificity indicates that eosinophil might be a real target of the immune derangement like the decreased lymphocytes (Sun et al., 2020). Differently, the modest value of sensitivity might be explained by a certain degree of heterogeneity between severe COVID-19 patients, as demonstrated by immunophenotyping studies that identified different clusters of the cytokine storm signature (Lucas et al., 2020; Mathew et al., 2020). There is a statistically significant difference between peripheral eosinophils’ blood count in non-severe and severe patients, pointing out that this population undergoes conditioning during the acute phase of severe infection (Sun et al., 2020; Xie et al., 2020).

Nevertheless, basophils and eosinophils should contribute to the antiviral response and could complicate the immunopathology. These cells undergo a dynamic change during severe disease: they increase from acute to recovery phases (Rodriguez et al., 2020). Other authors analyzed in more detail eosinophil cell populations. These populations exhibit a temporary growth of CD62L + eosinophils from day 2 to day 6 after hospital admission (Rodriguez et al., 2020). Expansion of CD62L + eosinophils seems to be attributed to IFN-γ, one of the most relevant cytokines in severe COVID-19, and the IFN-γ levels show an increase together with the increment of CD62L + eosinophils (Rodriguez et al., 2020). The specific phenotype of such eosinophils apparently belongs to a population of lung-resident eosinophils rather than to circulating eosinophils induced by the inflammation response, and CD62L + pulmonary-resident eosinophils have an important role in the organization of inflammatory responses in the lung (Mesnil et al., 2016). Rodriguez et al. (2020) think that the clonal growth of CD62L + eosinophils, which occurs after the development of a severe pulmonary immunopathology (one week after hospital admission) is correlated to the hyper-inflammation of the lungs in COVID-19 patients (Rodriguez et al., 2020).

In a recent post-mortem series of SARS-CoV-2 deceased patients, eosinophils were found in the alveolar *interstitium* (Damiani et al., 2020). In a case report, pulmonary eosinophilic vasculitis (with transmural eosinophilic infiltrate) was found in a severe COVID-19 patient that underwent bronchopulmonary lavage and lung biopsy on day 32 after intubation. No allergic disorder was previously known. BALF showed 36% eosinophils and 2.4 pg/ml IL-5. After two weeks of corticosteroid treatment, a subsequent bronchoalveolar lavage was made that showed 3% eosinophils and 2.3 pg/ml IL-5 (Luecke et al., 2021). Another case report described a clinical picture of eosinophilic pneumonia in a COVID-19 patient, diagnosed by increased eosinophils in BALF, which responded well to steroid treatment (Murao et al., 2020). However, it must be pointed out that the findings of eosinophils in severe COVID-19 lungs do not directly demonstrate that they are responsible for the damage. The role of eosinophils in pneumonia’s immunopathology still needs to be fully understood. Another point favoring eosinophils’ involvement is that skin dermatoses have been described in COVID-19 patients, in which increased eosinophils were found (Gianotti et al., 2020). The preferential expansion of lung-resident eosinophil is not in contrast with the observation that, in the most severe COVID-19 patients, peripheral blood count of eosinophils is generally decreased. Noteworthy, eosinopenia might depend on the migration of circulating eosinophils from the peripheral blood to the infected organs (Azkur et al., 2020).

## Severe Asthma in COVID-19 Patients: A Case-Study

Bronchial asthma is divided into two major phenotypes, which are characterized by Th2-high (eosinophilic) and Th2-low (non-eosinophilic) immune responses (Kuruvilla et al., 2019). There is still a debate in the scientific literature if patients with bronchial asthma would be at increased risk of developing a severe COVID-19 form and relative admission to the intensive care unit (Avdeev et al., 2020; Williamson et al., 2020; Choi H. G. et al., 2020). Until now, there are limited data about the effective risk of severe COVID-19 course in the population of asthmatic patients (Kow et al., 2020). A possible explanation because asthma does not appear to be a relevant risk factor for COVID-19 has been reported by Jackson et al. (2020) (Jackson et al., 2020). They hypothesized that atopic patients express lower levels of the *ACE2* gene in their airways. In fact, SARS-CoV-2 uses the ACE2 receptor to infect the host’s cells. Asthmatic children with allergen sensitization showed a progressive ACE2 decrease in the nasal epithelium. Similar results were reported in adults with mild asthma that received allergen provocation (Jackson et al., 2020).

Furthermore, a position paper from European Allergologists and Clinical Immunologists’ leading societies highlights that there is currently no evidence for an increased risk of a severe COVID-19 course in allergic patients (Klimek et al., 2020). This statement is particularly surprising as asthma exacerbations can usually be triggered by respiratory infections (Flores-Torres et al., 2019). This interesting fact has been confirmed in different countries such as China, the USA, South Korea, and Italy (Klimek et al., 2020; Zhu et al., 2020). In detail, in Wuhan, the percentage of seriously ill or deceased COVID-19 patients with known bronchial asthma was far below the prevalence of asthma (Li et al., 2020). In a real-world observational study performed using administrative data from Korea, 7,590 confirmed SARS-CoV-2 infection were identified. Among them, 218 (2.9%) had asthma. The mortality rate was higher in asthmatic patients than non-asthmatic controls (7.8 vs. 2.8%), but after adjusting for age, sex, and underlying conditions, asthma reveals not to be a significant risk factor for mortality (OR, 1.317; 95% CI, 0.708–2.451). Indeed, none of the asthma treatments (including ICS, inhaled corticosteroids; LABA, long-acting β2-agonists; LAMA, long-acting muscarinic antagonists; LTRA, leukotriene receptor antagonists; SABA, short-acting β2-agonists) influences the mortality rate or admission to ICU in multivariate analysis and even asthma’s severity was not associated with higher mortality (Choi Y. J. et al., 2020). These results were similar to those reported by another study from Daegu, Korea (Kim et al., 2020). Other authors demonstrated that asthma diagnosis was not associated with worse outcomes among severe COVID-19 patients 65 years or younger hospitalized within the New York City area, without considering age, obesity, or other high-risk comorbidities (Lovinsky-Desir et al., 2020). Other studies carried out in Italy confirm that the proportion of asthmatic patients in hospitalized COVID-19 positive patients is very small and suggest that asthma itself cannot be considered an independent risk factor for COVID 19 (Caminati et al., 2021) and does not appear to be one of the most relevant risk factors for ICU admission (Grasselli et al., 2020). There are several possible explanations for these findings. One explanation is that asthmatic patients are usually treated with systemic or inhaled corticosteroids, which have been demonstrated to ameliorate the disease course in severe COVID-19 (Peters et al., 2020; Rogliani et al., 2020; Sterne et al., 2020). Moreover, many severe asthmatic patients are under treatment with biologicals that inhibit type 2 immune responses via various mechanisms. The cited position paper does not dissuade to avoid such biological therapies but emphasizes that the potential effects of biologicals on the immune response in COVID-19 are currently unknown.

The first reports show that the disease course is not worse in COVID-19 patients with eosinophilic diseases under biological therapy, compared to those infected patients with eosinophilic disorder not treated with biologicals (Heffler et al., 2020). The pharmacological blockade of type 2 inflammation by therapeutic antibodies against IgE, IL-5, or IL-5/IL-4/-13 receptors, so far has not been suspected to increase the risk of viral infections, also in the respective approving clinical trials (Klimek et al., 2020). For example, in the Italian Severe Asthma Network (SANI) cohort, 26 patients received a confirmed (11) or a suspected diagnosis of COVID-19 (15). 21 patients with COVID-19 used biologicals: 15 (71%) anti-IL-5 or anti-IL5R drugs (mepolizumab n = 13; benralizumab n = 2) and 6 (29%) anti-IgE drug (omalizumab). In this small population, all patients were treated with inhaled corticosteroids/long-acting β2-agonists, and only two patients deceased. The SANI registry cohort includes 1,504 patients, 65% of them receive biological treatments (anti-IL5 or anti-IL5R drugs: 52.9%, anti-IgE: 47.1%). In their large cohort of severe asthmatics, few COVID-19 diagnoses were made. The mortality rate among severe asthmatic patients with a SARS-CoV-2 diagnosis was 7.7%, lower than that recorded in the Italian population (14.5%). Among the severe asthmatic group with COVID-19, the majority were treated with anti-IL5 drugs (71%), with a minority with anti-IgE (29%) (Heffler et al., 2020). These preliminary data suggest that severe asthma subjects are not at higher risk of SARS-CoV-2 infection or development of a severe COVID-19 form of the disease.

Similar results were derived from the Belgian registry of Severe Asthma. Fourteen severe asthmatic patients with a SARS-CoV-2-confirmed infection were collected from the registry cohort; five of them were hospitalized and none of them displayed asthma exacerbation, required systemic treatment with corticosteroids, invasive ventilation, or death. Only three patients received oxygen supplementation. Additionally, there was no difference in the incidence of SARS-CoV-2 infection between asthmatic patients who were on treatment with biologic therapy (four patients received anti-IgE and seven patients received anti-IL-5 or anti-IL-5R) and asthmatic patients not on treatment with biologics (Hanon et al., 2020). On the other hand, nine patients from the Dutch Severe Asthma Registry under biological treatment received a diagnosis of COVID-19, seven of them were hospitalized, and five entered to ICU. Six patients were on anti-IL5 therapy and three of them were admitted to ICU (all three patients were obese). Finally, only one patient died, but he had obesity and diabetes, known comorbidities for fatal outcome (Eger et al., 2020). Other case reports do not indicate a worse clinical course in COVID-19 patients exposed to anti-IgE omalizumab (Lommatzsch et al., 2020) and, anti-IL-5R benralizumab (Renner et al., 2020a; Renner et al., 2020b). Although only a few cases were reported, it can be speculated that biological inhibitors of type 2 response can have a possible impact on aberrant immune response, and thus can protect infected subjects from severe complications of COVID-19.

Besides the potential protective effects of asthma medications, it must be pointed out that a possible explanation for a limited prevalence of asthma in COVID-19 patients might be because asthmatic patients are more aware of the greater risk of exacerbations of their condition, and thus have paid more attention to hygiene prescriptions and have been even more protected than the general population.

On the other hand, several reports challenge the hypothesis that severely asthmatic patients do not display a higher risk for severe COVID-19 (Choi H. G. et al., 2020; Williamson et al., 2020; Zhu et al., 2020); therefore, further studies focused on different asthma phenotypes are needed to better understand the association between asthma and COVID-19 severity.

## Discussion

To date, there are five monoclonal antibodies addressed against type 2 immune activation authorized as a specific target therapy for the treatment of severe eosinophilic asthma (Chaplin, 2020):

‐ omalizumab, which binds to IgE, blocking their interaction to the relative IgE receptor on basophils and mast cells; it down‐regulates the expression of IgE receptor (MacGlashan et al., 1997);
‐ benralizumab, an IL‐5 receptor antagonist, which is expressed on the surface of eosinophils and basophils, provoking their apoptosis (Kolbeck et al., 2010);
‐ mepolizumab, targeted to IL‐5, blocking the binding with the respective receptor expressed on eosinophils;
‐ reslizumab, targeted to IL‐5, blocking the binding with the respective receptor expressed on eosinophils;
‐ dupilumab, which slows down type 2 inflammation, blocking IL‐4 and IL‐13;

The hypothesis presented here is based on the observation that in severe COVID-19 patients, Th2 immune response is stimulated and eosinophils may play a central role in precipitating immune derangement and aggravating SARS-CoV-2-induced pneumonia. Because IL-5 is essential for the survival, maturation, and activation of eosinophils, it is suggested that IL-5 inhibitor drugs might block eosinophils activation in severe COVID-19 patients.

Immunophenotyping studies showed a moderate increase of eosinophils in severe COVID-19 patients, at least in a subset of them, with a mean blood count of around 100 cells/μL (Lucas et al., 2020; Mathew et al., 2020). Anti-IL-5 drugs (benralizumab, mepolizumab, reslizumab) are currently indicated as an add-on therapy for subjects with severe eosinophilic asthma, not responding to standard treatments, diagnosed with a peripheral blood eosinophils count of 150 cells/μL or higher at the beginning of treatment (GlaxoSmithKline, 2015; Teva, 2016; AstraZeneca, 2018). So, the question that arises is why such drugs should be beneficial in COVID-19 patients, especially those most severely affected.

In the EMA’s Summary of Product Characteristic of mepolizumab, a combined analysis of the MEA112997 (DREAM) and MEA115588 (MENSA) approving trials is reported. Mepolizumab given at 75 mg IV/100 mg s.c. provided a significant reduction rate of clinically asthma exacerbations when given to patients with severe refractory eosinophilic asthma with a baseline blood eosinophil count as low as <150 cells/μL, with a reduction of exacerbation rate of 0.67. The effect size was larger, with an increasing count of eosinophils (GlaxoSmithKline, 2015). Mepolizumab reduces blood eosinophils and decreases the active migration of these cells to the lungs after stimulation with an allergen, but has a limited effect on respiratory resident eosinophils in asthma (Johansson et al., 2013; Kelly et al., 2017).

IL-5 receptor α (IL-5Rα) has been found in bronchial epithelial cells and allows epithelial barrier maintenance (Barretto et al., 2020). IL-5 beneficial effects on a mouse model of influenza do not depend only on eosinophils. Hence, IL-5Rα is expressed on migrated neutrophils in the lungs and neutrophils from other tissues (Gorski et al., 2019). IL-5Rα on activated neutrophils can promote signal transduction and, when activated by low concentrations of IL-5, causes a reduction of ROS production (Gorski et al., 2019). Children with asthma exacerbation exhibit both neutrophils and eosinophils recruitment and activation (Norzila et al., 2000). Interestingly, patients with mild asthma and rhinovirus infection that received mepolizumab treatment displayed a lower increase of neutrophils and neutrophil-derived myeloperoxidase in both BALF and sputum but also an increment of B lymphocytes and secretory IgA (Sabogal Piñeros et al., 2019b).

The role of neutrophils during SARS-CoV-2 infection is currently under investigation (Borges et al., 2020; Tomar et al., 2020; Wang et al., 2020). Specifically, the peripheral blood count of neutrophils is significantly higher in severe COVID-19 patients than those with moderate disease and can be considered a prognostic factor for a severe course (OR 1.5, 95% CI: 1.0–2.1) (Wang et al., 2020). Moreover, neutrophils count increases within 7–9 days since symptoms onset and correlate with radiologic findings (Wang et al., 2020). Activated neutrophils drive the production of neutrophil extracellular traps (NET) composed of DNA and toxic proteins that lead to cell death (named NETosis) and tissue damage (Cheng and Palaniyar, 2013). Transcriptome analysis conducted in COVID-19 patients showed up-regulation of NET-associated genes. Thus, neutrophils and NETs can contribute to immunopathology in infected lungs (Wang et al., 2020). Anti-IL-5 therapy might block neutrophils’ contribution to COVID-19 pneumonia.

IL-5Rα has been found in B-cell progenitors and activated B cells. On B cells, IL-5 stimulation participates in the plasma cell differentiation process (Takatsu, 2011). However, mepolizumab promotes the activation of the antiviral immune response, like NK cells potentiation, B lymphocytes’ survival, and IgA secretion (Contoli and Papi, 2019). Patients receiving mepolizumab should experience higher viral replication, so it should not be given in the first phase of the infection, like corticosteroids (Contoli and Papi, 2019).

Considering that eosinophils may participate in COVID-19’s immunopathology and that anti-IL-5 drugs could be effective even starting from relatively modest levels of eosinophil counts, it is tempting to speculate that treating COVID-19 patients with such biologicals might prove beneficial. Because eosinophils participate in antiviral immune response, anti-IL-5 drugs should not be administered in patients in the first stage, characterised by viral replication and limited inflammation. In such a setting, these monoclonal antibodies might be detrimental, similarly to corticosteroids (Siddiqi and Mehra, 2020). High-risk patients with hypoxia in the second stage of the disease [namely pulmonary phase IIB according to Siddiqi et al. proposal (Siddiqi and Mehra, 2020)] might be the best setting to try using anti-IL-5 biologics and prevent eosinophils recruitment. This assumption is based on the observations made by Lucas et al. (2020), who showed an increase of IL-5 levels within 6–10 days from symptoms onset, and a subsequent increase of eosinophils count on 11–15 days from symptoms onset (Lucas et al., 2020). The clinical context is similar to that of the RECOVERY trial, whereby dexamethasone efficacy was demonstrated: namely patients who require oxygen therapy (from supplemental oxygen to mechanical ventilation) (Sterne et al., 2020; Horby et al., 2020). Consistently, the efficacy of dexamethasone on COVID-19 mortality maybe also due to eosinophils apoptosis induction (Ilmarinen et al., 2014).

It is predicted that hospitalised COVID-19 patients at risk of fatal outcome should be treated with anti-IL-5 drugs as soon as possible before peripheral eosinophil count falls. Clinical risk scores aiming to predict intensive care admission or death are still under investigation (Galloway et al., 2020), and the timing of changes in leucocyte counts (including that of eosinophils) is yet to be precisely determined in COVID-19.

An idea of the effect of IL-5 antagonism in severe COVID-19 may be inferred from the results of a small clinical trial that explored granulocyte-macrophage colony-stimulating factor (GM-CSF) antagonism (De Luca et al., 2020). GM-CSF is a growth factor produced by macrophages, T-cells, epithelial cells, endothelial cells, and fibroblasts: it promotes the survival of monocytes, the differentiation of macrophages, and the activation of T cells subpopulations (Bonaventura et al., 2020). GM-CSF facilitates the migration of eosinophils in the lung and promotes their survival, especially in a setting of allergic inflammation (Nobs et al., 2019). Mavrilimumab, a monoclonal antibody that blocks GM-CSF, has been shown to improve clinical outcomes compared to standard care in hospitalized patients (De Luca et al., 2020).

## Conclusion

A reasoned timing and appropriate patient selection in a randomized controlled clinical trial is the only way to establish whether IL-5 antagonism in COVID-19 is beneficial or harmful. The second and early third stages of the disease, with high-risk moderate and severe patients, respectively, should be the appropriate setting to try using IL-5 drugs.
